# Neutrophil Recruitment and Leukocyte Response Following Focused Ultrasound and Microbubble Mediated Blood-Brain Barrier Treatments

**DOI:** 10.1176/appi.focus.20104

**Published:** 2022-01-25

**Authors:** Charissa Poon, Carly Pellow, Kullervo Hynynen

**Affiliations:** Physical Sciences Platform, Sunnybrook Research Institute, Toronto, Ontario, Canada; Institute of Biomedical Engineering, University of Toronto, Toronto, Ontario, Canada;Department of Medical Biophysics, University of Toronto, Toronto, Ontario, Canada.

## Abstract

(Appeared originally in Theranostics 2021; 11:1655–1671)

Reprinted under Creative Commons Attribution License

